# Trial characteristics, methods and reported challenges of decentralised clinical trials: a scoping review

**DOI:** 10.1136/bmjopen-2025-106823

**Published:** 2025-11-21

**Authors:** Alexandra Cussen, Katherine Littler

**Affiliations:** 1 Adolescent Medicine, The Royal Children’s Hospital Melbourne, Parkville, Victoria, Australia; 2 The University of Melbourne Department of Paediatrics, Parkville, Victoria, Australia; 3 Murdoch Children’s Research Institute Clinical Sciences Theme, Parkville, Victoria, Australia; 4 Research for Health, World Health Organization, Geneva, Switzerland

**Keywords:** Clinical Trial, Digital Technology, Review, Research Design, STATISTICS & RESEARCH METHODS

## Abstract

**Abstract:**

**Objectives:**

To map the landscape of decentralised clinical trials (DCTs) by summarising characteristics, methods and reported challenges of published DCTs.

**Design:**

Scoping review, reported according to the Preferred Reporting Items for Systematic Reviews and Meta-Analyses for Scoping Reviews (PRISMA-ScR) checklist.

**Data sources:**

Ovid MEDLINE and PubMed were searched through to 21 August 2024.

**Eligibility criteria:**

We included reports of completed DCTs (defined as a trial of an intervention, with a comparison arm, in which some or all trial activities occurred away from the trial centre). All intervention types were included.

**Data extraction and synthesis:**

A single reviewer extracted data to a structured extraction sheet. Descriptive statistics (frequencies) are reported for study characteristics and the terminology used to describe trial methods. Decentralised methods used were coded separately for each trial stage.

**Results:**

53 papers met inclusion criteria. Most studies (34/53) were conducted in the USA. Mental health (18 studies) and COVID-19 (11 studies) were the predominant research areas. 24 (of 53) studies investigated pharmaceutical interventions, while others examined nutritional interventions, medical devices and behavioural interventions. Recruitment, screening and consent were commonly conducted remotely. A range of methods, including online, in-person and telemedicine, was used to collect outcome measures. Several studies experienced challenges related to participant retention and biased recruitment. Terminology regarding decentralisation was inconsistent across studies.

**Conclusions:**

DCTs are rapidly increasing in use, and commonly cited advantages include reduced costs and reduced participant burden. This review identifies key research areas using DCTs and highlights a need for standardised terminology, comprehensive reporting of methods and limitations, and robust regulatory frameworks. Development of formal ethical and reporting standards is essential to ensure effective and responsible implementation of DCTs in clinical research.

**Strengths and limitations of this study**

- This study provides a comprehensive overview of published decentralised clinical trials (DCTs), summarising the current landscape including global distribution, trial types, intervention types, sponsorship and decentralised methods used.
- This review summarises the reported limitations in completed DCTs, providing real-world data on challenges encountered with the use of decentralised methods.
- Terminology in the description of decentralised methods is known to be inconsistent; therefore, it is possible that our search did not identify all relevant studies.

## Introduction

 Clinical trials evaluate the safety and efficacy of medical interventions, including medications, vaccines, medical devices and behavioural interventions. While there is no single globally recognised definition of decentralised clinical trials (DCTs), they are most often considered to be clinical trials in which some or all trial-related activities occur at locations other than traditional clinical trial sites, for example, within participants’ homes, local medical clinics or local pathology laboratories.^1^,^3^ DCTs are also referred to as remote, virtual or web-based trials, although the specific term ‘DCT’ has increased in use since 2018.^1^ Various stages of the trial may be decentralised, including recruitment, screening, consent processes, delivery of the intervention, follow-up visits and collection of outcome measures.^2 4 5^ DCTs often use digital and web-based technology to facilitate remote trial activities. Digital tools include online recruitment, electronic consent, telemedicine appointments and wearable monitoring devices.^2 4 5^ Trials may take a hybrid form (some activities take place at the trial site and some remotely) or may be fully decentralised (participants do not visit the trial site at any point during the trial).^2^ In the context of the COVID-19 pandemic from 2020 onwards, DCTs became a rapidly growing area in clinical trials, as restrictions would otherwise have forced active trials to be paused or cancelled.^6 7^ Increased availability of digital technology has also facilitated the remote conduct of trials.^2 4^

DCTs may offer practical and ethical advantages over traditional site-based trial designs. They may also pose challenges that are not experienced in site-based trials. DCTs are often described as participant-centric and less burdensome for participants.^8^ While participant convenience is a stated aim, DCTs are also convenient, lower-cost and less resource-intensive for trial sponsors.^9^ DCTs may improve participant recruitment and retention due to lower burden of participation.^10^,^13^ DCTs may be useful to study medical conditions that render travel and mobility difficult for patients (such as neurodegenerative conditions), as well as rare diseases, for which it may be difficult to recruit enough participants within proximity of a trial site.^14 15^ While a potential advantage of DCTs is improved equity in trial participation, online recruitment and participation methods may result in biased recruitment, including exclusion of groups who do not have reliable access to required digital technologies or failure to overcome more complex barriers to participation.^12 13^ DCTs may also involve lower costs for participants (both time and financial costs) and increased flexibility by adapting trial activities to the participant’s own schedule.^8^ Digital technologies (such as wearable devices and smartphone applications) allow for real-time, continuous, objective safety monitoring. This may improve accuracy and speed of adverse effect reporting; however, it may also increase incidental findings and pose issues for secure data storage and appropriate use of the large amount of data generated.^16^ Self-collected and self-reported data may be subject to issues related to data validity and quality, reporting bias and incomplete or missing data.^16^,^18^

Existing regulatory documents emphasise the need for risk–benefit evaluation prior to choosing a decentralised trial model.^19^,^21^ Despite the increasing frequency of DCTs, there has been limited focus on the emerging ethical issues associated with these trial designs.^16 18^ In this article, we reviewed the characteristics and reported limitations of completed, published DCTs, in order to summarise the research landscape of DCTs to inform the development of ethical guidance.

### Aims

This scoping review aimed to summarise the global distribution, key research areas, methodology and reported limitations and challenges of completed, published DCTs.

## Methods

### Data sources and searches

PubMed and Ovid MEDLINE were searched on 16 August 2024 and 21 August 2024 for combinations of the terms “decentralised” or “decentralized” or “remote” or “virtual” and “trial” or “clinical trial”. The full search strategies are described in online supplemental file 1. The search was limited to studies published between 1 January 2014 and 16^h^ August 2024. Although use of the term “decentralised” has increased in the literature since 2018,^1^ the trial widely cited as the “first” DCT was published in 2014.^22^ This review aimed largely to map the landscape of completed, published DCTs in order to inform ethical guidance development; inclusion of results from the preceding 10 years back to the time of the first recognised DCT was determined to be useful in order to achieve a more complete picture of the literature. The results of initial searches were supplemented by snowballing (reference lists from the papers identified in the initial search were searched and relevant papers were added for review). If a study protocol was found in the search but the full study was not found, ClinicalTrials.gov, PubMed and Google Scholar were searched for the published report of the completed study, which was then included if the study met criteria for full-text inclusion (see below).

### Study selection

Data were extracted from published reports of completed DCTs. Studies were included if they met the criterion of being a report of a completed DCT (defined as a trial of an intervention, with a comparison arm, in which some or all trial activities occurred away from the trial centre). All intervention types were included, including pharmaceutical, medical device, psychological, behavioural or health promotion interventions.

Feasibility studies, pilot studies, study protocols with no associated report of a completed trial, and trials with no comparison arm (‘single-arm’ trials) were excluded. Studies that aimed to compare in-person data collection or treatment delivery methods with remote methods (rather than being a clinical trial of an intervention) were excluded—for example, a study that compared clinical outcomes between patients who were followed up by telehealth visits compared with those followed up by in-person clinic visits would be excluded.

### Screening

All citations were imported into EndNote V.21, and duplicates were removed. Citations were then uploaded to Covidence.org (a web-based systematic review software tool). Title and abstracts were screened, full-text articles were then retrieved and screened for inclusion. Where the first reviewer was uncertain regarding inclusion, papers were assessed by the second reviewer. No automated tools were used for screening.

### Data extraction and analysis

Data extraction was completed using a structured spreadsheet (Microsoft Excel). The extraction sheet was developed based on the aims of the review to include relevant data points regarding trial characteristics (box 1). These data points were agreed between the authors and prefilled to the sheet prior to data extraction. The first five papers (10%) were used to pilot the extraction sheet and finalise coding of categorical variables (eg, sponsor and Institutional Review Board (IRB) subcategories). A meeting was held between the reviewers following this pilot phase to finalise the coding framework. If the required data were missing from the published trial report, data were sought from the published study protocol and/or the trial registration page on ClinicalTrials.gov where available.

Box 1 **Data points extracted**

1. Author, year.
2. Location of trial (country/jurisdiction).
3. Types of research using decentralised trials.
  - Trial type (eg, phase 3 medication trial).
  - Disease/condition.
  - Sponsor (eg, hospital or university, industry, government).
  - IRB/Research Ethics Committee (REC) oversight type (eg, central/institutional).
4. Terminology: term used in the title or abstract to describe trial methods (eg, decentralised, remote, web-based).
5. Decentralised methods used (eg, remote recruitment, delivery of study materials to participants’ homes).
6. Reported challenges or limitations arising from the use of decentralised methods.

Descriptive statistics (frequencies) were reported for study characteristics and the terminology used to describe trial methods (points 1–3 in box 1). These data are presented in text and summary tables. For the analysis of decentralised methods used, the five publications related to the ACTIV-6 trial (see below) were treated as one study (n=49 rather than n=53) due to identical methodology in each publication. To describe trial methods used, each trial stage was coded separately (eg, recruitment, screening, consent, etc) and categorised as either decentralised, in-person or hybrid. The challenges and limitations reported by study authors were categorised thematically with categories refined through discussion between reviewers: a narrative synthesis of these data is presented in the text.

### Patient and public involvement

None.

## Results

**Figure 1:**
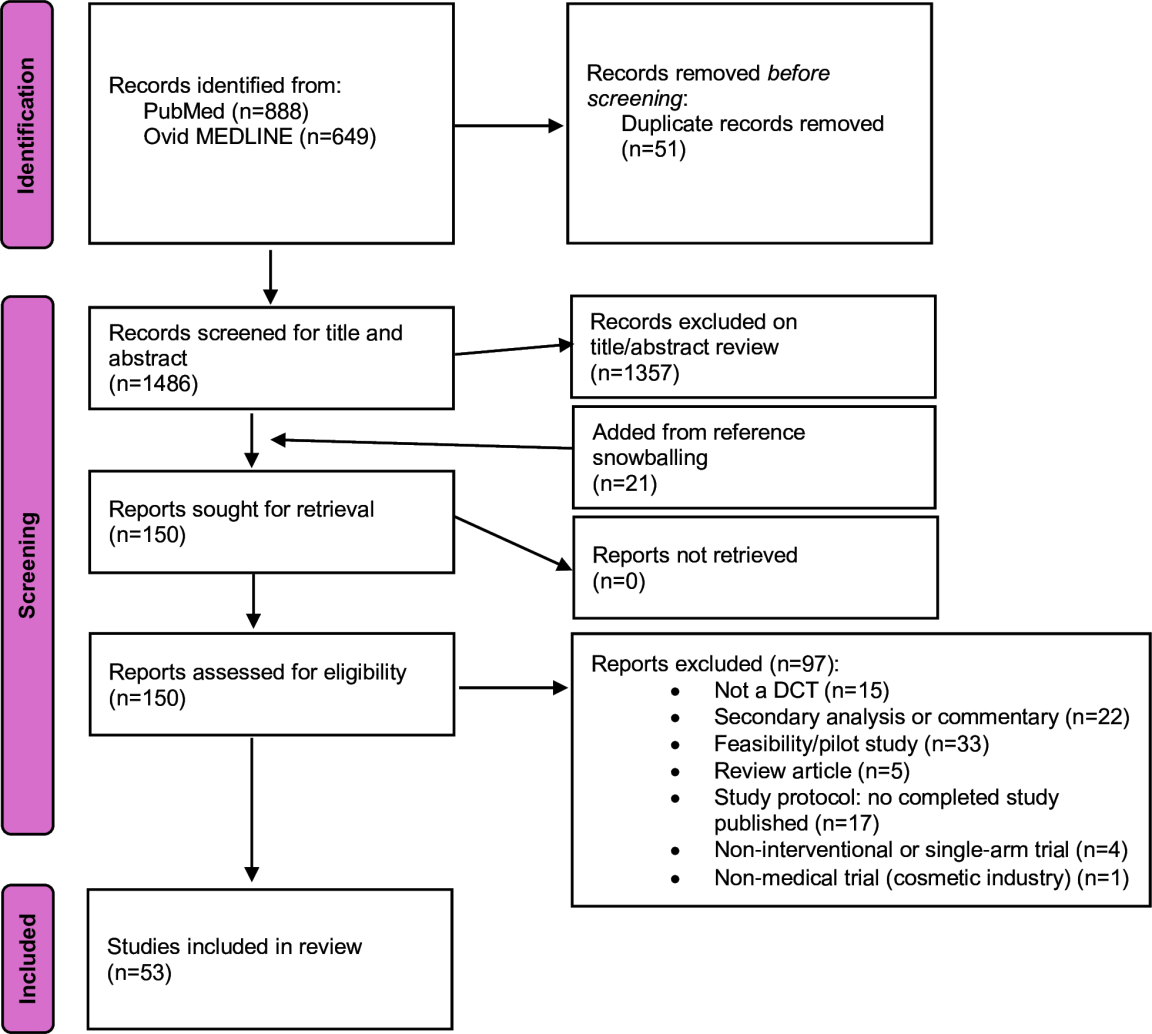
Preferred Reporting Items for Systematic Reviews and Meta-Analyses (PRISMA) flow diagram. DCT, decentralised clinical trial.

Our database searches identified 1537 articles. Following removal of duplicates, 1486 articles underwent title and abstract screening, resulting in 129 articles eligible for full-text review. A further 21 articles were added to full-text screening from reference snowballing. Following full-text review, 53 papers met inclusion criteria for data extraction and analysis (figure 1).

### Study characteristics

Table 1 and online supplemental table summarise characteristics of the included studies. Most studies were conducted in the USA (34 out of 53 publications).^22^,^55^ Five of these publications were related to one large, multicentre trial (the ACTIV-6 trial) that was conducted in the USA during the COVID-19 pandemic.^25 35 37 38 46^ There were five studies from the UK,^56^,^60^ three studies from Australia^61^,^63^ and two each from Japan^64 65^ and China.^66 67^ There was one study each from Canada,^68^ Denmark,^69^ Germany,^70^ Korea,^71^ New Zealand^72^ and Sweden.^73^ There was one multicountry study within Europe.^74^

**Table 1:** Summary of study characteristics

Study characteristic	Number of papers (total n=53)
Location of trial	
USA^22^,^55^	34
UK^56^,^60^	5
Australia^61^,^63^	3
China^66 67^	2
Japan^64 65^	2
Canada^68^	1
Denmark^69^	1
Germany^70^	1
Korea^71^	1
New Zealand^72^	1
Sweden^73^	1
Multiple countries (within Europe)^74^	1
Condition group	
Mental health^2324 28 32 36 40 50 52^,^54 60 63^	18
COVID-19 infection^25^,^2731 33 35 37^	11
Dermatology^44 58 69 70 72^	5
Cardiovascular^30 45 56 57^	4
Health promotion/ public health^34 43 49 51^	4
Oncology^29 47^	2
Gastroenterology^62 75^	2
Gynaecology^48^	1
Infectious diseases^67^	1
Nephrology^71^	1
Neurology^41^	1
Neuroscience^42^	1
Osteoarthritis^61^	1
Urology^22^	1
Intervention type	
Pharmaceutical^2225^,^27 30^	24
Online or via mobile application^2324 28 34 36 40 43 47 49^,^54 60 63^	22
Nutritional^62 71^	2
Medical device^48 70^	2
Medication reminders^41^	1
Exercise^29^	1
Infection self-testing kits^67^	1
Sponsor	
Hospital/university^2326^,^31 33 40 41 43 47 49^	29
Industry^22 24 32 34 39 42 44 45 48 52 62 69 70 72^	14
Government or national institute^3663^,^65 71^	5
Principal investigator^25 35 37 38 46^	5
Ethics approval	
Hospital or university Institutional Review Board (IRB) 2328 29 31 33 40,43 47 49	26
Central private IRB^24 27 32 34 36 45 48^	7
Central public IRB^56^,^5969 72 73^	7
Multiple (eg, multisite/mix of public and private)^2225 26 30 35 37^,^39 44 46 74^	11
Not mentioned^70 71^	2

The most studied condition groups were mental health (18 papers^2324 28 32 36 40 50 52^,^54 60 63^) and COVID-19 (11 papers^25^,^2731 33 35 37^). Five studies were in dermatology.^44 58 69 70 72^ Four studies each were in cardiovascular conditions^30 45 56 57^ and health promotion/public health.^34 43 49 51^ There were two oncology studies^29 47^ and two gastroenterology studies.^62 75^ The remaining studies were in infectious diseases,^67^ neuroscience,^42^ neurology,^41^ nephrology,^71^ osteoarthritis,^61^ urology^22^ and gynaecology.^48^

The intervention in 24 studies was related to medications (including drug trials,^2225^,^27 31 33 35 37^ trials comparing medication dose or timing^30 57^ and trials of supplement or complementary medicines^32 42 61 72^). Of the 53 studies, 22 described a behavioural or mental health intervention delivered online or via a mobile application,^2324 28 34 36 40 43 47 49^,^54 60 63^ and 2 described nutritional interventions.^62 71^ In the other studies, the interventions were a medical device,^48 70^ an electronic pill-bottle for medication reminders,^41^ provision of infection self-testing kits^67^ and an exercise intervention.^29^

Of the 53 publications, 29 were sponsored by hospital or university sponsors,^2326^,^31 33 40 41 43 47 49^ 14 by industry^22 24 32 34 39 42 44 45 48 52 62 69 70 72^ and 5 by government or national institutes.^3663^,^65 71^ The ACTIV-6 trial (five papers) listed the principal investigator as the trial sponsor.^25 35 37 38 46^

Ethics approval was obtained from a single hospital or university institutional review board in 26 of 53 studies,^2328 29 31 33 40^,^43 47 49^ a single central private review board in 7 studies,^24 27 32 34 36 45 48^ a central or regional public review board in 7 studies,^56^,^5969 72 73^ and by more than one review board in 11 studies.^2225 26 30 35 37^,^39 44 46 74^ Two studies did not mention ethics approval.^70 71^

### Terminology

With regard to terminology to describe the use of decentralised methods, 21 studies used the term “decentralised” (or “decentralized”).^24^,^2629 31^ Other terms used included “web-based”,^22 51 53 74^ “online”,^54^ “internet-based”,^61 68^ “mail-based”,^56^ “remote”,^23 36 40 42 45 48^ “virtual”,^39 41 48 73^ “digital”^50^ and “hybrid”.^69^ Some studies were labelled as decentralised but used hybrid methodology (eg, an in-person option at the trial site was available for some stages of the trial).^25 31 35 37 38 46 71^ Several studies mentioned the use of decentralised methods (eg, telehealth visits or social media recruitment) in the abstract but did not use a specific term to describe these methods.^49 52 55 58 59 63^ For seven studies, there was no mention of “decentralised” or another related term in the title or abstract.^27 28 30 47 60 67 72^ These studies were included because on full-text review, use of decentralised methods was described.

### Decentralised methods used

**Figure 2:**
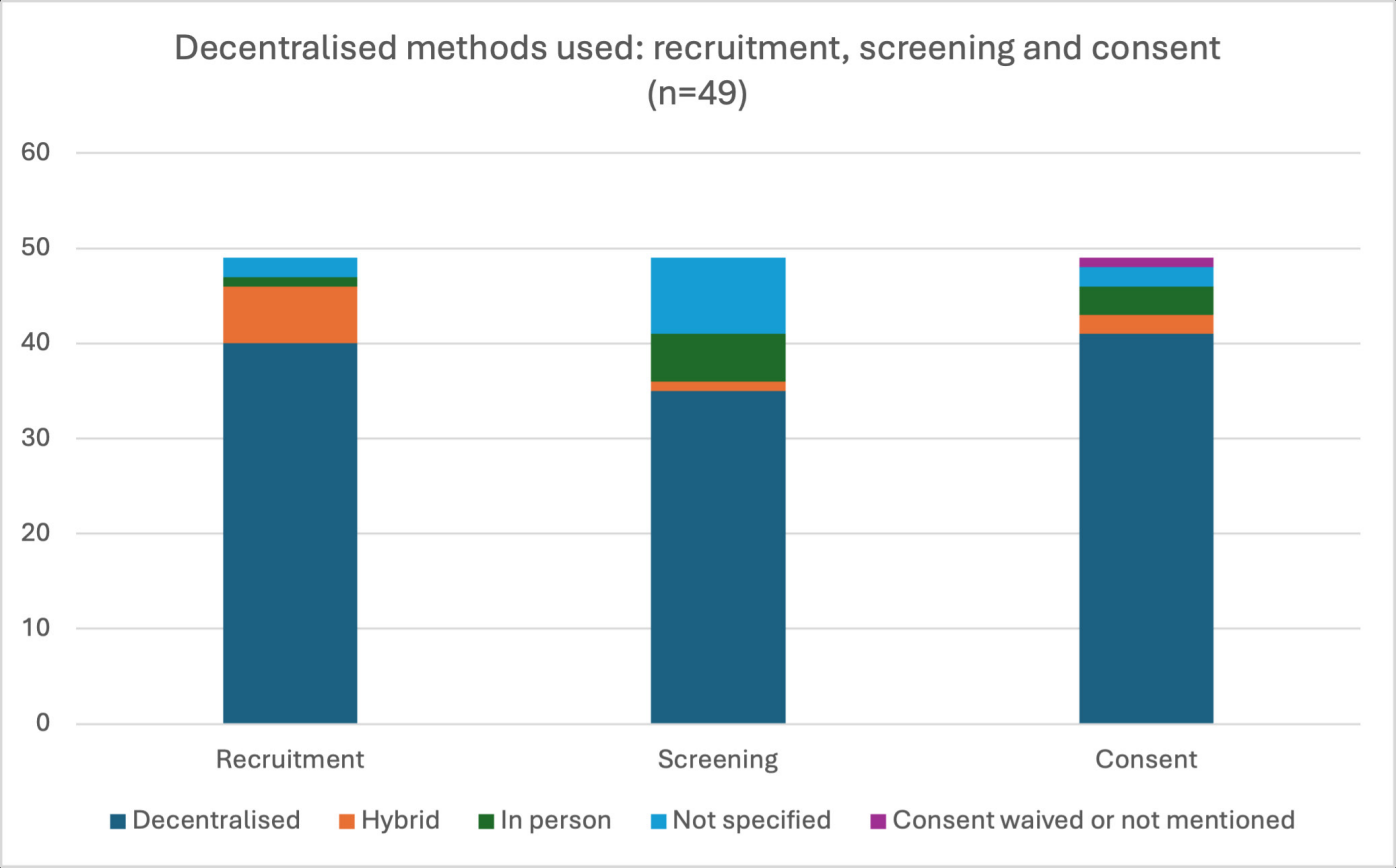
Decentralised methods used: recruitment, screening and consent (n=49).

The five papers related to the ACTIV-6 trial were counted once for these analyses to avoid skewing data, because the methods described in each of the five papers are the same. Therefore, 49 papers are included for analysis of decentralised methods used. Figure 2 shows a summary of methods used for recruitment, screening and consent processes.

For recruitment, of 49 papers, 40 used decentralised methods, 6 used hybrid methods, one study recruited in-person and methods were not specified in 2 papers. Decentralised methods included recruitment via websites, social media, targeted email lists or searches of electronic health records for participants who may be eligible.^23 56 62^ Of the 49 studies, 35 used decentralised methods to screen participants, one used hybrid screening, 5 screened in-person and 8 did not specify their screening methods. Where hybrid recruitment methods were used, some participants were recruited or screened online and some in-person (eg, within on-site clinics).^25 31 35 37 38 41 46^

Informed consent was collected electronically (‘eConsent’) in 41 of 49 studies; 2 studies used hybrid methods, 3 studies collected consent in- person, and 2 studies did not mention consent method. One study, conducted via a mobile application, stated that consent was waived as participants had agreed at the time of downloading the application that their data may be used for research.^34^ Seven studies, all of which studied an online or mobile application intervention, involved participants under the age of 18 years.^2428 49 51^,^54 63 68^ There was variation in whether parental consent was obtained or waived, and the age for which parental consent was waived also varied between studies. Consent methods included using remote/telemedicine consultations to explain the study, a prerecorded video to explain the study, or a written description of the study that participants read online. Electronic signatures were commonly used.

**Figure 3:**
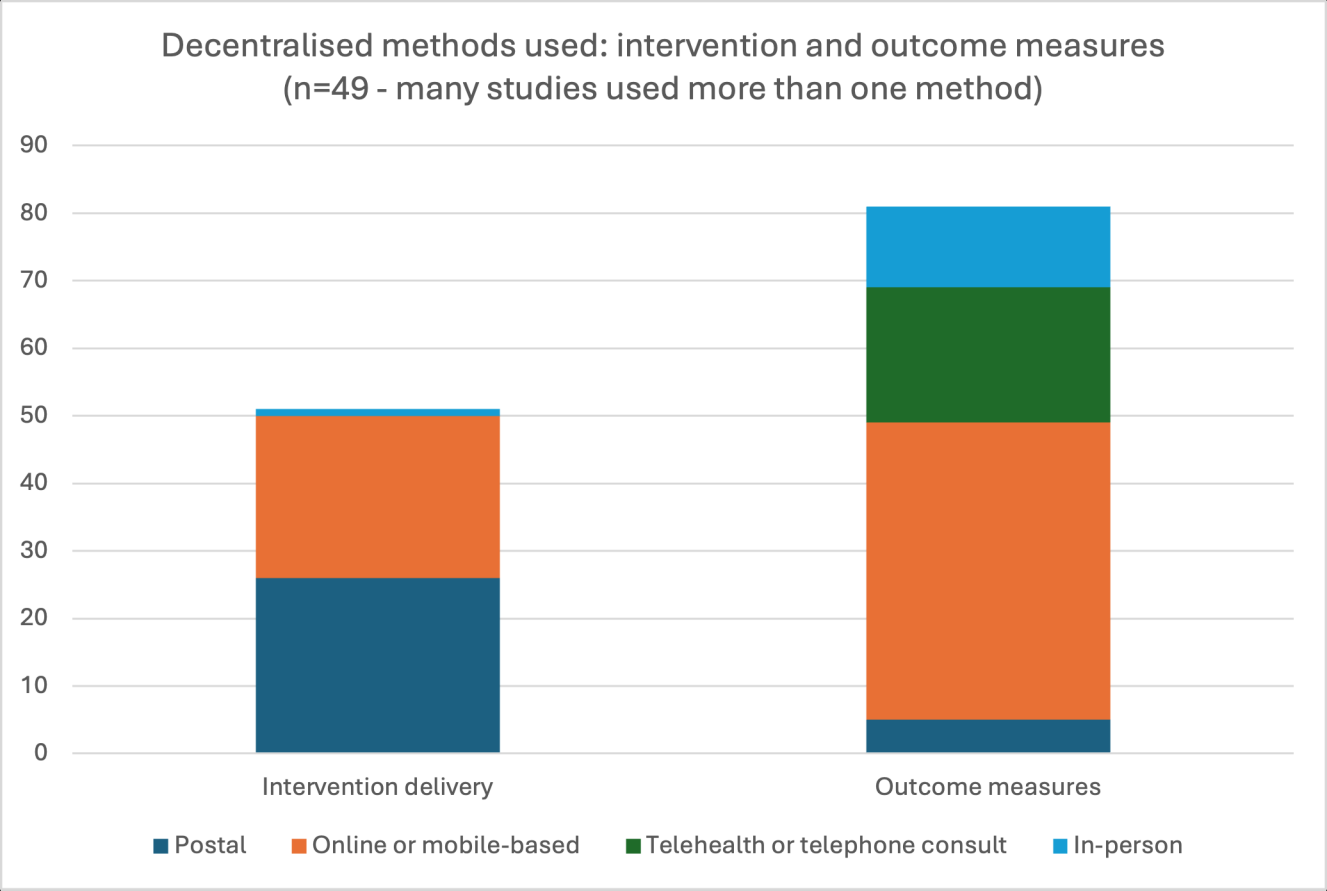
Decentralised methods used: intervention and outcome measures (n=49; many studies used more than one method).

Figures3  4 show a summary of decentralised methods used for administration of the intervention and collection of outcome measures. For the administration of the intervention, 26 studies involved postal or courier delivery of intervention material to participants’ homes. Interventions delivered to participants’ homes included pharmaceuticals in 20 studies (prepared at a central pharmacy then sent by post or courier),^2225^,^27 31^ food or preprepared meals,^62 71^ medical devices,^48^ self-testing kits,^67^ electronic pill-bottles^41^ and exercise equipment^29^ (figure 3). In four studies, patients used their own supply of medication or obtained medication in-person from a local pharmacy.^30 56 57 72^ Of 49 studies, 24 involved an intervention delivered online or via a mobile application, for example, a single-session mental health or an exercise intervention delivered by online video (figure 3).^2324 28 29 34 36 40 43 47 49^,^54 60 63^ Most of the mental health studies were conducted entirely online or via mobile applications, with no mention of any contact between study staff and participants throughout the trial.^2324 28 36 40 50 52^,^54 60 63 66 74^

**Figure 4:**
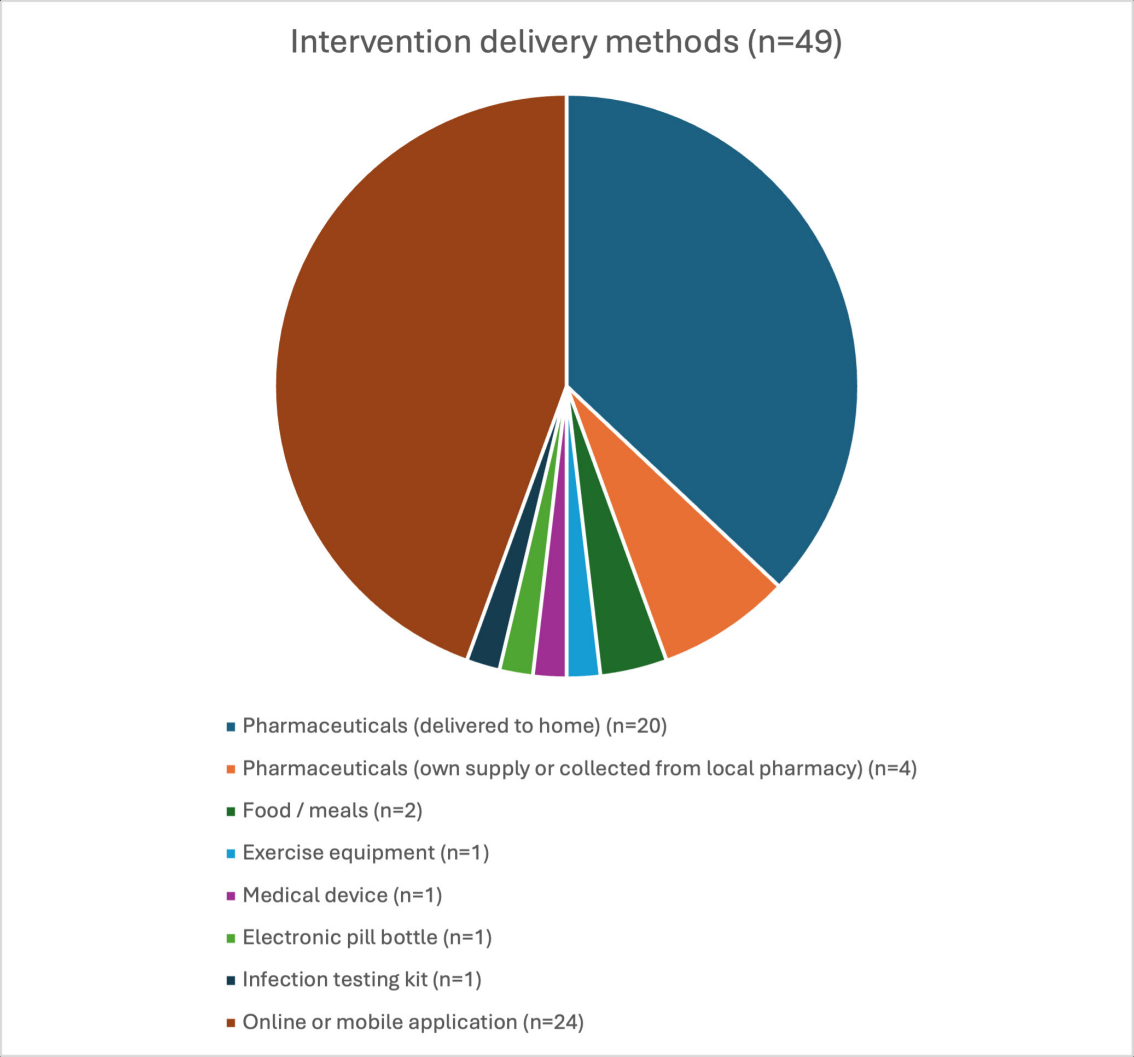
Intervention delivery methods (n=49).

Outcome measures were collected by a variety of decentralised methods, which can be broadly categorised into online/mobile application, postal, video/telephone and in-person (at a trial centre, local facility or within participants’ homes). Many studies used more than one decentralised method to collect outcome measures. Online or mobile application methods included self-reported data entry via an online patient portal, website or mobile application. Some studies collected continuous data via wearable devices (eg, heart rate measurement via a FitBit).^45^ Postal methods include return of questionnaires, unused medications or samples via courier or post.^41 47 56^ Telemedicine or telephone methods included replacing in-person study visits with video or telephone consultations, or collection of questionnaire data over the telephone.^25^,^2730 31 39 41 44 48 64 69^ In-person methods at the participants’ home included participant self-collection of data (eg, blood pressure readings^57^), self-collection of swabs, samples or fingerprick blood tests by participants,^62 67^ or study staff visiting the participant’s home to conduct assessments^44^. Some in-person visits occurred via local pathology laboratories or the patient’s usual local care provider.^22 33^

18 studies mentioned the decentralised methods used to facilitate participant retention, which included text messages, emails, reminder telephone calls or notifications via a mobile telephone application to remind participants to complete outcome measures.^2324 26 27 31 33 40^,^43 45 60 63^ 25 studies mentioned methods used to monitor adverse events (AEs).^2225^,^27 31 33 35 37 38 43^ These included self-reporting via a mobile application, collection of AE reports as part of online outcome-measure questionnaires, and telephone contact from study staff at regular intervals to specifically ask about AEs.^25 26 35^

### Reported challenges arising from the use of decentralised methods

Of the 53 included studies, 14 did not discuss any limitations or challenges arising from their use of decentralised methods. Of those that did discuss limitations, these can be divided into challenges experienced within the study, and theoretical limitations that were discussed but not necessarily experienced.

With regard to challenges experienced, the ACTIV-6 trial studies (drug trials for COVID-19 infection in which the drugs were delivered directly to participants’ homes) cited delays in receipt of the trial drug (typically 5–6 days after the onset of viral symptoms).^25 35 37 38 46^ Three studies encountered inaccurate or missing data due to issues with home monitoring devices or technology connection issues.^27 41 42^ 19 of 53 studies had difficulties with participant retention and high attrition or drop-out rates.^22^,^2428 32 34 40 49 50 52^ These were mostly digital mental health studies. However, the REMOTE trial, published in 2014, frequently cited as ‘the first entirely web-based trial conducted under an IND application’, experienced such significant drop-out that confirmation of safety and efficacy of the drug was precluded.^22^ Six studies, in addition to the ACTIV-6 trial, mentioned difficulty recruiting a representative sample.^24 28 34 54 63 66^ One hybrid study stated that ‘hidden (financial) costs’ of decentralised methods should be taken into account (including the costs of technology and the need to have a decentralised research team available to assess safety concerns).^69^ Seven studies in addition to the ACTIV-6 trial papers described that they did not achieve recruitment of a representative sample.^24 52 54 55 63 66 68^

With regard to theoretical limitations, a further five studies discussed possible biased recruitment related to the need to have access to and/or confidence using technology, and subsequent impacts on generalisability.^36 61 64 65 74^ One study discussed the possibility of ‘deception’ in recruitment: when participants were recruited online, self-report of the diagnosis required for participation could not be verified.^64^ Several studies discussed possible data quality issues for participant self-reported or self-collected data.^34 41 43 47 57 60 61 66 67 74^ One study mentioned lack of physical examination as a possible limitation.^48^ One study discussed possible data quality issues related to differences in expertise between home-visit staff and expert clinicians located at the trial site.^44^ One study noted that decentralised studies may result in a ‘transfer of burden’ from trial sites to participants or remote staff.^43^

## Discussion

This scoping review of published DCTs summarises characteristics, methods and reported methodological issues arising from these trials. By including all types of interventions and sponsorships, this review presents a more complete picture of the landscape of completed DCTs than other recent review articles and cross-sectional analyses of clinical trial databases.^4 5 76^ This review identified 53 studies. Most studies were undertaken in the USA, and most were in mental health and COVID-19 research. There was a mix of public and private sponsorship, and of institutional and central ethical review board use. Two studies did not mention ethical review, and several studies of young people aged under 18 years did not obtain parental consent. There was a range of interventions used. 49 studies were included for analysis of methods used (due to 5/53 studies being part of the same large multisite trial). In 24 of 49 studies, the intervention was medication related. Other interventions included nutritional interventions, medical devices, health-related technologies and online behavioural and mental health interventions. Terminology to describe decentralised trial activities was inconsistent, and in 7 of 53 studies, there was no mention in the title or abstract that decentralised methods had been used. 14 studies did not discuss any potential limitations or ethical issues related to the use of decentralised methods in their trial. However, high rates of participant drop-out and difficulties with participant retention were common, especially in online mental health studies. Studies also described possible biased recruitment limiting generalisability and a high reliance on self-collected or self-reported data.

Our finding that most DCTs were undertaken in the USA is consistent with other recent review articles and cross-sectional analyses of clinical trial databases.^4 5 76^ There was some difference in the key medical condition groups represented in our review as compared with other studies. Studies of mental health conditions and behavioural interventions were included in our review, but were excluded from previous reviews of pharmaceutical interventions.^4 5 76^ Most of the mental health DCTs in our review involved behavioural interventions, delivered either online or via a mobile application.

Frequently cited advantages of DCTs include improved patient recruitment and retention, and inclusion of groups of participants usually excluded from research due to participation being less burdensome for participants.^10^,^1377^ In contrast to this, many studies included in our review reported difficulties with participant retention, high rates of drop-out, inadequate recruitment and recruitment of non-representative samples. This discrepancy between the theoretical benefits and real-world experience of DCTs may warrant further research, and methodological modifications may need to be considered to ensure that DCTs are representative, allow equitable inclusion and produce quality data.

Existing regulatory guidance and published literature on ethical issues in DCTs discuss many possible issues arising from the use of decentralised methods, including ensuring participant safety and well-being, considerations for delivery of medications and other products to participants’ homes and data security issues.^3 18 21^ It is therefore interesting that 14 studies did not discuss any potential limitations or issues related to the use of decentralised methods. This again highlights a need for clear ethical guidance and reporting criteria to ensure that DCTs can be undertaken safely, to maximise benefits to participants and minimise potential harms.

A strength of our study is that it presents a comprehensive picture of the DCT landscape, in contrast to other review articles and cross-sectional analyses of clinical trial databases that limited analyses to drug trials only,^4^ industry-sponsored trials only,^76^ or excluded trials for which the intervention was mental health or behavioural.^5^ We present data on decentralised trial methods that have been successfully used in trials that have continued to completion.

We note a lack of consistent terminology when reporting DCTs, which presents a challenge for reviews, analyses and guidance development, as studies may be difficult to identify and may be missed when using keyword searches. These findings are consistent with a 2023 review article of terms used to describe clinical trials, which found significant heterogeneity in terminology and called for consensus and the adoption of a single term to describe these trials.^1^ Future guidance should include reporting guidelines, and future research should consistently and clearly report the use of decentralised methods. A limitation of our review is that, given inconsistent use of terminology, some studies may have been missed, including those that did not mention the use of decentralised methods in the title or abstract. This review does not claim to be truly comprehensive in identifying all decentralised trials that do not use the term. For example, many trials in low- and middle-income settings (such as malaria trials in Africa) have used practices now considered decentralised, long before the concept of DCT evolved into its current understanding.^78^ The term ‘Active Case Detection’ has been used for decades to describe in-home testing, treatment and interviews as part of malaria clinical trials. ^78^ This limitation can be seen in the geographic spread of trials identified in this article: most of identified studies were from the United States which may relate to the recent increase in use of the term ‘DCT’. Future guidance should include reporting guidelines, and future research should consistently and clearly report the use of decentralised methods in the published trial report. Our study may also be subject to publication bias, as studies that encountered significant issues may not have been completed and published. This may be particularly relevant when identifying methodological and ethical challenges.

## Conclusions

The use of decentralised methods in clinical trials is rapidly increasing. The COVID-19 pandemic and increased availability of digital technologies may have contributed to this increase. This scoping review of published DCTs reveals some advantages and challenges of this emerging approach. While DCTs offer theoretical advantages in improving trial accessibility and reducing participant burden, we found that many studies experienced difficulties with participant retention. The predominance of trials in high-resource settings highlights the need to consider implementation in diverse contexts. Inconsistent terminology and variable reporting of decentralised methods were evident, presenting a challenge for systematic evaluation and guidance development. A surprisingly high number of published trials did not discuss potential challenges or ethical considerations of their use of decentralised methods. This suggests a need for more rigorous attention to these aspects. These findings highlight the importance of developing standardised terminology, comprehensive reporting requirements and robust ethical frameworks to guide future implementation of DCTs.

## Supplementary material

10.1136/bmjopen-2025-106823 online supplemental file 1

10.1136/bmjopen-2025-106823 online supplemental table 1

## Data Availability

Data sharing not applicable as no datasets generated and/or analysed for this study.
