# Supplementary material for: Trial characteristics, methods and reported challenges of decentralised clinical trials: a scoping review
Source: BMJ Open. 2025 Nov 21;15(11):e106823. doi: 10.1136/bmjopen-2025-106823 (PMC12658491; doi:10.1136/bmjopen-2025-106823)
Supplement: online supplemental file 1 [file bmjopen-15-11-s001.docx]

Supplementary File 1: full search strategies including any filters or limits used.

| Ovid MEDLINE  21^st^ August 2024  649 results  *Exported to Endnote 21* |
| --- |
| 1. (decentrali* or virtual* or remote).tw,kf. 2. virtual.mp. 3. remote.mp. 4. decentralised.mp. 5. clinical trial/ 6. 1 OR 2 OR 3 OR 4 7. 5 AND 6 8. limit 7 to yr=“2014 – Current” |

| PubMed  16^th^ August 2024 & 21^st^ August 2024  888 results  *Exported to Endnote 21* |
| --- |
| "decentralised clinical trial"[tiab:~3] OR "decentralized clinical trial"[tiab:~3] OR “decentralised trial”[tiab:~3] OR “decentralized trial” [tiab:~3] OR "virtual trial"[tiab:~3] OR "virtual clinical trial"[tiab:~3] OR "remote trial"[tiab:~3] OR "remote clinical trial"[tiab:~3]  limited to 2014 - Current |
