# Supplementary material for: Trial characteristics, methods and reported challenges of decentralised clinical trials: a scoping review
Source: BMJ Open. 2025 Nov 21;15(11):e106823. doi: 10.1136/bmjopen-2025-106823 (PMC12658491; doi:10.1136/bmjopen-2025-106823)
Supplement: online supplemental table 1 [file bmjopen-15-11-s002.docx]

### Supplementary Table. Characteristics of included publications

| Author (Year) | Country/Region | Trial type | Disease / Condition | Sponsor | IRB/REC oversight type |
| --- | --- | --- | --- | --- | --- |
| Akechi et al (2024) [65] | Japan | Online or mobile intervention | Mental health / Oncology | Government/ National Institute | Institutional (University) |
| Akechi et al (2022) [66] | Japan | Online or mobile intervention | Mental health / Oncology | Government/ National Institute | Institutional (University) |
| Arean et al (2016) [24] | United States | Online or mobile intervention | Mental health | Hospital/University | Institutional (University) |
| Arnaud et al (2016) [75] | Sweden, German, Belgium, Czech Republic | Online or mobile intervention | Mental health | Hospital/University | Multiple: institutional and central |
| ASCEND  Study Collaborative group [57] | United Kingdom | Phase 4 drug | Cardiovascular | Hospital/University | Central - NHS |
| Boucher et al (2024) [25] | United States | Online or mobile intervention | Mental health | Industry | Private |
| Boulware et al (2023) [26] | United States | Phase 3 drug | Covid-19 | Principal Investigator | Multiple: institutional (Hospital) |
| Bramante et al (2023) [27] | United States | Phase 3 drug | Covid-19 | Hospital/University | Multiple: Institutional and Private |
| Bramante et al (2022) [28] | United States | Phase 3 drug | Covid-19 | Hospital/University | Private |
| Cairns et al (2022) [56] | United States | Phase 2 drug | Covid-19 | Hospital / University | Institutional (University) |
| Chung et al (2024) [72] | Korea | Nutritional intervention | Renal | Government/ National Institute | Not mentioned |
| Dahne et al (2023) [51] | United States | Online or mobile intervention | Mental health | Hospital/University | Institutional (University) |
| Dobias et al (2021) [29] | United States | Online or mobile intervention | Mental health | Hospital/University | Institutional (University) |
| Ghaderi et al (2020) [74] | Sweden | Online or mobile intervention | Mental health | Hospital/University | Regional (public) |
| Greene et al (2020) [53] | United States | Online or mobile intervention | Mental health | Industry | Institutional (University) |
| Hawkey et al (2022) [60] | United Kingdom | Medication | Gastroenterology | Hospital/University | Regional (public) |
| Hillhouse et al (2017) [52] | United states | Online or mobile intervention | Public health | Hospital/University | Institutional (University) |
| Jones et al (2024) [30] | United States | Exercise intervention | Oncology | Hospital/University | Institutional (Hospital) |
| Jones et al (2021) [31] | United States | Medication dosing | Cardiovascular | Hospital/University | Multiple: Institutional |
| Kaizer et al (2023) [32] | United States | Phase 2 drug | Covid-19 | Hospital/University | Institutional (University) |
| Kolobaric et al (2023) [33] | United States | Complementary medicine | Mental health | Industry | Private |
| Krumholz et al (2024) [34] | United States | Phase 2 drug | Covid-19 | Hospital/University | Institutional (University) |
| Liu et al (2021) [62] | Australia | Phase 2/3 (Complementary medicine) | Osteoarthritis | Hospital/University | Institutional (University) |
| Lu et al (2023) [67] | China | Online or mobile intervention | Mental health | Hospital/University | Institutional (University) |
| Mackenzie et al (2022) [58] | United Kingdom | Medication timing | Cardiovascular | Hospital/University | Central – NHS |
| Marshall et al (2022) [35] | United States | Online or mobile intervention | Health promotion | Industry | Private |
| Martin et al (2023) [71] | Germany | Device/technology | Dermatology | Industry | Not mentioned |
| McCarthy et al (2023) [36] | United States | Phase 3 drug | Covid-19 | Principal Investigator | Multiple: Institutional |
| Moreno et al (2021) [50] | United States | Online or mobile intervention | Public health | Hospital/University | Institutional (University) |
| Muench et al (2023) [37] | United States | Online or mobile intervention | Mental health | Government/ National Institute | Private |
| Naggie et al (2022) [38] | United States | Phase 3 drug | Covid-19 | Principal Investigator | Multiple: Institutional |
| Naggie et al (2022) [39] | United States | Phase 3 drug | Covid-19 | Principal Investigator | Multiple: Institutional |
| O’Dea et al (2020) [64] | Australia | Online or mobile intervention | Mental health | Government/ National Institute | Institutional (University) |
| Orri et al (2014) [23] | United States | Phase 4 drug | Urology | Industry | Multiple: central (private) and institutional (University) |
| Ortner et al (2024) [70] | Denmark | Phase 1/2a drug | Dermatology | Industry | Central - Capital Region of Denmark |
| Piazza et al (2023) [40] | United States | Phase 3 drug | Covid-19 | Industry | Multiple: central (private) and institutional |
| Pratap et al (2018) [41] | United States | Online or mobile intervention | Mental health | Hospital/University | Institutional (University) |
| Radomski et al (2020) | Canada | Online or mobile intervention | Mental health | Hospital/University | Institutional (University) |
| Rice et al (2021) [42] | United States | Device/technology | Neurology | Hospital/University | Institutional (Hospital) |
| Robinson et al (2024) [43] | United States | Complementary medicine | Neuroscience | Industry | Institutional (University) |
| Santer et al (2023) [59] | United Kingdom | Phase 3 drug | Dermatology | Hospital/University | Regional (public) |
| Schleider et al (2022) [55] | United States | Online or mobile intervention | Mental health | Hospital/University | Institutional (University) |
| Schwinn et al (2015) [54] | United States | Online or mobile intervention | Mental health | Hospital/University | Institutional (University) |
| Seixas et al (2023) [44] | United States | Online or mobile intervention | Health promotion | Hospital/University | Institutional (Hospital) |
| Shafran et al (2019) [61] | United Kingdom | Online or mobile intervention | Mental health | Hospital/University | Institutional (University) |
| Shortt et al (2022) [73] | New Zealand | Complementary medicine | Dermatology | Industry | Central (public) |
| Silverberg et al (2024) [45] | United States | Phase 2a drug | Dermatology | Industry | Multiple: Institutional |
| Spertus et al (2022) [46] | United States | Phase 3 drug | Cardiovascular | Industry | Private |
| Stewart et al (2023) [47] | United States | Phase 3 drug | Covid-19 | Principal Investigator | Multiple: Institutional |
| Volk et al (2020) [48] | United States | Online or mobile intervention | Oncology | Hospital/University | Institutional (University) |
| Wang et al (2022) [68] | China | Testing intervention | Infectious Diseases | Hospital/University | Institutional (University) |
| Wang et al (2024) [63] | Australia | Nutritional intervention | Gastroenterology | Industry | Institutional (Research institute) |
| Weinstein et al (2022) [49] | United States | Device/technology | Gynaecology | Industry | Private |
