# Supplementary material for: Trial characteristics, methods and reported challenges of decentralised clinical trials: a scoping review
Source: BMJ Open. 2025 Nov 21;15(11):e106823. doi: 10.1136/bmjopen-2025-106823 (PMC12658491; doi:10.1136/bmjopen-2025-106823)
Supplement: Supplementary file 3 [file bmjopen-2025-106823.reviewer_comments.pdf]

PEER REVIEW HISTORY

BMJ Open publishes all reviews undertaken for accepted manuscripts. Reviewers are asked to complete a checklist review form and are provided with free text boxes to elaborate on their assessment. These free text comments are reproduced below.

ARTICLE DETAILS

Title (Provisional)

Trial characteristics, methods, and reported challenges of decentralised clinical trials: a scoping review

Authors

Cussen, Alexandra; Littler, Katherine

VERSION 1 - REVIEW

**Reviewer**	**1**
**Name**	**Love, Sharon**
**Affiliation**	**MRC CTU at UCL, MRC Clinical Trials Unit at UCL**
**Date**	**26-Aug-2025**
**COI**	**None**

This research is using scoping review methodology to summarise published decentralised clinical trials. The study is carried out well and the results described clearly. This paper would be a useful addition to the literature.

Major comment

The Prisma-ScR checklist says the full search strategy for at least one of the data bases is on page 3. To me a full search strategy is what I would type in whereas this is not what is given. Page 4 does not to me have the details of extraction sheet testing. Page3-4 does not have enough detail of how data is handled and analysed.

Minor comments

Condition groups only reports 50 out of the 53 in the text and 51/53 in table 2. I think the other 2 or 3 need to be accounted for.

Table 1 Radomski et al (top of page 16) has not got a ref number. Whilst I think table 1 should be included, I wonder if it should be in a supplementary file.

Maybe in the paper it would be useful to have tables showing which papers had which attributes.

Figure 1 – I don't currently understand the number in the screening section. Records screened for title and abstract = 1486 taking away those excluded =1336, gives 150 papers. 21 are added in from snowballing, yet the number is still 150 (I was expected 150+21=171)

In the discussion on page 11, the term "many" is used as a summary of 14/53 studies. "Many studies did not discuss any potential limitations or ethical issues related to the use". Can you find a different summary word (less than many). Maybe give the number? Maybe say "surprisingly high". This happens again at the top of page 12. And in the conclusion on page 12.

Typos

Page 9 "Many studies used a more than one decentralised method to collect outcome measures." Need to delete the "a"

Reviewer **2**

Name **Dettwiller, Pascale**

Affiliation **University of South Australia Allied Health & Human Performance Academic Unit, Department of Rural Health**

Date **31-Aug-2025**

COI **None**

Very valuable article in a research space where standardisation is required.

VERSION 1 - AUTHOR RESPONSE

Reviewer: 1
Dr. Sharon Love, MRC CTU at UCL
Comments to the Author:
This research is using scoping review methodology to summarise published decentralised clinical trials. The study is carried out well and the results described clearly. This paper would be a useful addition to the literature.

Major comment
The Prisma-ScR checklist says the full search strategy for at least one of the data bases is on page 3. To me a full search strategy is what I would type in whereas this is not what is given. Page 4 does not to me have the details of extraction sheet testing. Page3-4 does not have enough detail of how data is handled and analysed.

- We have now included our full search strategy as Supplementary File 1. We have cited the supplementary file in the main text Methods section where the search strategy is described.
- We have now included additional detail in the Methods section under the heading "Data extraction and analysis" specifying data extraction sheet testing, handling of duplicate papers, handling of categorical variables and thematic categorisation where applicable.

Minor comments
Condition groups only reports 50 out of the 53 in the text and 51/53 in table 2. I think the other 2 or 3 need to be accounted for.
Thank you, this was an error and has now been amended in both the text and table.

Table 1 Radomski et al (top of page 16) has not got a ref number. Whilst I think table 1 should be included, I wonder if it should be in a supplementary file.
Maybe in the paper it would be useful to have tables showing which papers had which attributes.

- Reference number for Radomski et al now added to Table 1.
- Table 1 moved to a supplementary file.
- We agree that Table 2 is more useful with inclusion of details of which papers had which attributes. We have now added the relevant references throughout Table 2.

Figure 1 – I don't currently understand the number in the screening section. Records screened for title and abstract = 1486 taking away those excluded =1336, gives 150 papers. 21 are added in from snowballing, yet the number is still 150 (I was expected 150+21=171)

- Thank you, this was an error in the flowchart figure- we have now amended the figure. The numbers included in the text were correct.
- 1357 papers were excluded on title/abstract review, 129 papers were identified from title and abstract screening + 21 added from snowballing = 150 papers underwent full text screening.

In the discussion on page 11, the term "many" is used as a summary of 14/53 studies. "Many studies did not discuss any potential limitations or ethical issues related to the use". Can you find a different summary word (less than many). Maybe give the number? Maybe say "surprisingly high". This happens again at the top of page 12. And in the conclusion on page 12.
We have now removed the word "many" and replaced with the number fourteen within the text (two instances). We have added the reviewer's suggested wording "A surprisingly high number" in the conclusion.

Typos
Page 9 "Many studies used a more than one decentralised method to collect outcome measures." Need to delete the "a"
Typo now amended.

Reviewer: 2
Dr. Pascale Dettwiller, University of South Australia Allied Health & Human Performance Academic Unit
Comments to the Author:
Very valuable article in a research space where standardisation is required.

*** ***

VERSION 2 - REVIEW

**Reviewer**	**1**
**Name**	**Love, Sharon**
**Affiliation**	**MRC CTU at UCL, MRC Clinical Trials Unit at UCL**
**Date**	**19-Sep-2025**

COI

Thank you for updating
